# Dental Societies

**Published:** 1882-04

**Authors:** 


### DENTAL SOCIETIES.

Editor Register.---Those who are in the habit of going to dental society meetings cannot help noticing what a small proportion of the practicing dentists attend them. Why is this thus? Societies unquestionably do a great deal in the way of disseminating knowledge. Such being the case, what excuse can a man find for staying away? Is it for fear some *gas bag* may occupy the time for from a half to three-quarters of an hour at a stretch ventilating his ignorance? This, *alas!* is one of the almost necessary evils, and one that we will have to endure for a while, as no practical method of *squelching* such seems yet to have been devised. Or do the absentees flatter themselves that they can get all that is of importance from the printed reports of the proceedings as they appear in the different dental journals? If they do, they are laboring under a delusion, and the sooner they divest themselves of it the better for *them*, as it is utterly impossible to make a report that will give the reader an absolutely accurate idea of what was said and done during the session. Appliances and interesting specimens are exhibited, and modes of operating are illustrated on the blackboard that words are inadequate to describe. They are, consequently, lost to those that stay away. Even if it were possible to convey to their cerebrums a correct impression of what transpired they would still be the loosers, as the reports appear any time between six weeks and six months after the meeting was held, and he that attends has the use of any new idea that may have been advanced just that much sooner than he that does not. This to the young practitioners is a fact of vital importance, and although he may have to make a sacrifice in order to attend, yet he will find that he will gather in valuable information; and, perhaps, try his hand in a discussion, stirring up some of the old fossils, thereby stimulating himself to increased diligence in his studies. He will learn that *all* dentists do not, like wine, improve with age. K.

